# Neural Bases of Brand Reputation Effect on Extension Evaluation: An ERPs Study

**DOI:** 10.3389/fnins.2021.704459

**Published:** 2021-08-23

**Authors:** Chang Liu, Zhijie Song, Rui Shi

**Affiliations:** School of Economics and Management, Yanshan University, Qinhuangdao, China

**Keywords:** brand extension, reputation, fit, event-related potentials, corporate social responsibility, corporate ability

## Abstract

Brand extension, as a marketing strategy, is frequently utilized by enterprises to produce new products. There exist several critical factors determining its success, such as brand reputation and perceived fit. The present study adopts the event-related potentials (ERPs) method to explore the underlying neural mechanism of the joint influence of the two factors on consumers’ evaluation of brand extension. Specifically, consumers were presented with a brand with corporate social responsibility (CSR) or corporate ability (CA) reputation, following attached to an extension product (high fit vs. low fit). And then, they were given a 5-point scale to report their acceptance intention (AI) toward the brand extension. Behavioral data showed a higher AI and a shorter reaction time for high fit in contrast to low fit conditions. For low fit conditions, consumers were more inclined to accept the extension product with a brand with CSR than CA reputation. Neurophysiologically, CSR reputation evoked a larger P2 amplitude and LPP amplitude than CA reputation. Moreover, the low fit conditions elicited a more positive LPP amplitude than the high fit conditions in the context of a brand with a CSR reputation. Yet, for a brand with a CA reputation, the effect of perceived fit was not found. These results may reflect early attention resources engagement and altruistic motivation at the late stage during brand extension evaluation. The findings provided neurological evidence for which of the two types of brand reputation (CSR vs. CA) have a more positive effect on brand extension.

## Introduction

Brand extension is a popular and valuable strategy for leveraging the original brand names to establish new products (Swaminathan, 2003). Numerous brands widely adopt the strategy to attach multiple diverse product categories to extend corporates’ business range. For example, Mitsubishi, an automobile brand, launched electric vehicles; Quaker oatmeal is well known by the public, but recently, it broadened to milk powder areas; Pepsi-Cola even expanded from drink to fashion industries, introducing clothing and shoes. In a similar vein, most scholars have focused on the study of brand extension in the past 30 years and identified various factors of its success, such as the perceived fit between the parent brand and the extension product (Czellar, 2003), the prestige image of the parent brand (Eren-Erdogmus et al., 2018), the parent brand quality and reputation (Hur et al., 2013). Specifically, the perceived fit, which is often defined as category similarity between the original brand and extension product, is a crucial influencer of brand extension evaluations (Aaker and Keller, 1990; Herr et al., 1996). It has been pronounced by previous studies that the success of brand extension depends on how well the category of extension products near the parent brand (Völckner and Sattler, 2006; Sattler et al., 2010). They revealed that a brand with a high fit extension product was more acceptable by consumers than a low fit one. Even though the importance of near category extension with the brands has been widely underlined in the literature, there are certainly numerous examples of enterprises that have introduced distant extension products in the real market, both successfully and unsuccessfully. As such, recent studies have attempted to resolve how to improve consumer’s acceptance of low fit extension products (Parker et al., 2017; Shang et al., 2017; Zhang et al., 2020). For example, Parker et al. (2017) has provided insights into the suitable time of launching a brand’s first far extension product category. Zheng et al. (2019) and Zhang et al. (2020) have revealed the positive effect of distraction and the product display format on low fit extension evaluation.

In addition, brand reputation is also an essential determinant of brand extension evaluation (Wang et al., 2015), which is often considered as a formation of customers’ perspectives, impressions, and attitudes toward the enterprise (Fombrun and Shanley, 1990; Fombrun et al., 2000; Jung and Seock, 2016). Due to a favorable brand reputation is one of the most significant intangible assets driving corporate performance (Boyd et al., 2009), many companies adopt several strategies to enhance their reputation in a competitive market situation. For instance, brands always build a positive brand image and reputation through engaging in corporate social responsibility (CSR) activities or improving corporate ability (CA). Specifically, a brand with a good ability reputation signals high quality and continued innovation, while a socially responsible reputation connects with many social activities, such as philanthropic activities, community giving, and cause-related marketing (Brown and Dacin, 1997; Biehal and Sheinin, 2007). From the perspective of consumers, several researchers argue that a brand with strong ability will be more attractive than socially responsible (Johnson et al., 2017), whereas other researchers consider that consumers prefer brands with good socially responsible reputations (Hur et al., 2013). Moreover, a recent study by adopting a behavioral measurement has proved the essential role of the two types of brand reputation effect on brand extension (Johnson et al., 2019). They have indicated that the socially responsible reputation of a brand could provide a warm feeling for consumers and further effectively increase the acceptance of distant extension products. Nevertheless, the behavioral measurement method is limited to investigate consumers’ specific cognitive processes. In other words, it is necessary to apply neuroscientific tools for a better understanding of how the two types of brand reputation affect consumers’ mental process toward brand extension evaluation.

In terms of consumer neuroscience research, by using functional magnetic resonance imaging (fMRI), a few studies have provided evidence for consumers how to respond to CSR or CA-related messages at the brain level. For example, Couwenberg et al. (2017) demonstrated that functional (i.e., show good product quality) and experiential (i.e., warm expression) advertising evoked different brain areas, such as the temporal cortex and dorsolateral prefrontal cortex (DLPFC), related to lower- and higher-level cognitive processes. In addition, recent neuroimaging studies have captured the brain activities when subjects were exposed to prosocial brands (Lee, 2016), CSR messages (Medina et al., 2021), and green logo products (Lee et al., 2020). They found the activation of the anterior cingulate cortex (ACC) is involved in the evaluation of CSR-related messages, which was a brain region associated with improved attention and affective awareness (Seghier, 2013). Accordingly, it is natural to deduce that the processing of brand reputation during extension evaluation may likewise link to attentional and emotional mechanisms.

Event-related potentials (ERPs) are also one of the most efficient neural measurement tools, which hold a high temporal resolution and contribute to measuring consumers’ perceptual processing toward stimuli (Luck et al., 2000). Extensive research studies have revealed the consumers’ cognitive process of the evaluation of brand extension with different perceived fit levels by using the ERPs approach (Ma et al., 2007, 2008, 2014; Wang et al., 2012b; Jin et al., 2015; Fudali-Czyz et al., 2016; Yang and Kim, 2019). For instance, Ma et al. (2007, 2008) revealed the conflict effect or the categorization process when a brand extent to different category products. Recently, the brand extension literature in the neuroscience area start to focus on not only the one effect factor (e.g., perceived fit). Instead, they combine other determinants to investigate the consumers’ evaluation of brand extension jointly. Take for an example of Shang et al. (2017)’s study, they have found that the long-term memory conflict effect between the two brand strategies (brand logo vs. brand name strategy) and low fit extension product. Moreover, Ma et al. (2020) have added the brand familiarity factor into different category brand extension and suggested that consumers need to engage more cognitive resources in low familiar brand and high conflict extension product condition. However, the psychological process of consumers’ brand extension evaluation is not clear when they faced a brand with different types of reputation, such as CSR and CA. Furthermore, the neural basis of the joint effect of perceived fit and brand reputation on brand extension evaluation also remains a mystery. As a result, ERPs measurement was utilized to investigate consumers’ mental process toward brand extension, focusing on the two types of brand reputation (CSR vs. CA) and different perceived fit levels (high fit vs. low fit) in the present study.

Two vital ERPs components relevant to the study of brand extension are the early P2 component and the late positive potential (LPP) component. Previous studies have also verified that the two components are connected with attentional and emotional mechanisms (Zubair et al., 2020). Specifically, P2 is a positive potential that peaks approximately 200 ms after stimulus onset (Crowley and Colrain, 2004; Gonzalez-Villar et al., 2014). The P2 component has often been linked to attention-related processes, reflecting the early rapid automatic activity of attention engagement (Carretiea et al., 2000; Liu et al., 2010). In a study of the evaluation of brand extension, Ma et al. (2014) found that P2 could reflect the rapid and automatic assessment of attention resources and early low-level of category similarity processing between the brand-product word pair. Accordingly, the different perceived fit might have distinct influences on the P2 component of this study. Furthermore, considerable research studies have suggested that P2 are sensitive to emotional stimuli, and a pronounced P2 would emerge when subjects were presented with positive or negative rather than neutral stimuli (Bernat et al., 2001; Herbert et al., 2006). For example, Herbert et al. (2006) reported that the positive adjectives could engage more attention resources compared to neutral emotional adjectives, resulting in the P2 amplitude of the positive stimuli larger than that of the neutral stimuli. Guo et al. (2016) found that preference products could evoke relatively positive emotion, which was reflected in an increased positive P2 deflection for enhanced attention. Additionally, a recent study by Jing et al. (2019) found that subjects could distinguish which stimuli were more positive. They indicated that the deep discounts were more attractive and recruited more attentional resources, reflecting a greater P2 under deep discounts than shallow discounts conditions. In the current study, the two types of brand reputation (CSR and CA) are both positive stimuli for consumers. Notably, there is evidence that CSR activities can assist brands in presenting a warmer image (Kervyn et al., 2012), while a CA reputation provides a competence perspective (Aaker et al., 2012). Moreover, the perception of warmth and competence hold different emotional values (Ivens et al., 2015). Therefore, we suspected that a distinct P2 amplitude would appear under the two brand reputation conditions.

LPP, which belongs to the P3 family, is a late positive-going component that generally peaks at approximately 400–800 ms after presenting a stimulus with a maximum over the centro-parietal scalp (Schupp et al., 2000; Hua et al., 2014; Wang et al., 2019). It has been revealed that LPP is primarily associated with emotional arousals and motivational attention. High arousal and motivated stimuli will induce noticeable amplitudes of LPP (Hajcak et al., 2006; Handy et al., 2010). For example, Zhang et al. (2019) showed that the prominent logo aroused more significant motivations, and thus, a greater LPP amplitude was observed compared to inconspicuous ones of counterfeit luxury brands. Bosshard et al. (2016) found that brand attitude is related to emotional content, and liked brands would elicit a more positive LPP amplitude reflecting strong motivation levels. As a good brand reputation contribute to accumulating a positive brand attitude toward consumers (Ramesh et al., 2019), the two types of brand reputation events might evoke the LPP component. Moreover, Song et al. (2020) recently launched a brand extension study with a focus on prestige brands. They revealed the emotional transfer process of luxury brand extension evaluation and indicated that the high fit stimulus pairs evoked a strong arousing affect, and a larger LPP amplitude was induced in contrast to low fit conditions. Accordingly, we assumed that the different perceived fit between the given brand and product in the current study would elicit different amplitudes for LPP.

Overall, we hypothesize that the P2 and LPP components may emerge in different evaluation processing phrases of brand extension with the four conditions of brand reputation (CSR vs. CA) and perceived fit (high fit vs. low fit). In the neuromarketing domain, previous brand extension studies often adopt an S1-S2 paradigm to measure whether a brand (S1) extending to a new product or service (S2) is suitable or not (Ma et al., 2008; Yang et al., 2018; Song et al., 2020). But for the current experiment, a brand reputation event will be inserted into the stimuli of brand and extension product. To give participants a better understanding, we combined the S1-S2 paradigm and the stimuli presentation formation of a sentence conducting by Peng et al. (2015) and Liao et al. (2019). Therefore, a new paradigm was employed by the current study. Specifically, the subjects were first presented to a brand with a reputation event and then following a new product. In the meantime, there were two verbs used to connect brand, reputation event, and product for forming a sentence formation. Finally, we adopted a five-item scale to accurately investigate the consumers’ acceptance intention (AI) toward the given brand extension.

## Materials and Methods

### Participants

A total of 22 right-handed students from Yanshan University was invited to the current study. They had a normal or corrected-to-normal vision, and none of them had any history of neurological or psychiatric disorders. Prior to the formal experiments, written informed consent was obtained from all participants. This study was approved by the Internal Review Board of Yanshan University. All participants were paid ¥30 as a reward. Because of excessive ERP artifacts, two participants were rejected from the data set. And thus, data from 20 valid participants (6 males, age range: 19–24 years; Mage = 21.5 years, *SD* = 1.6) were for the following analysis.

### Materials

A 2 (brand reputation event: corporate social responsibility vs. brand ability) by 2 (extension fit: high fit vs. low fit) experiment was designed. We chose dairy brands as the stimuli materials for the following two reasons: on the one hand, dairy products exist in most Chinese people’s lives for a long time; on the other hand, consumers usually consider brand reputation factors in the choice of dairy brands (Ozdemir et al., 2020). Thus, eight Well-known native Chinese dairy brands including Yili, Mengniu, Huishan, and etc. were chosen from chinapp.com. Considering the individual difference, each participant was instructed to select four dairy brands from above eight brands they were familiar with, no preference and no hated as brand stimulus before the experiment. Second, six product names were selected from two different product categories (dairy and clothes) to constitute two types of extension fit levels with dairy brands. For high fit extension conditions, three dairy products (e.g., milk) were contained, as well as, for low fit extension stimuli, three clothes (e.g., T-shirt) were involved. Third, according to previous studies, a brand with a capable reputation signifies high quality and technology improvement, while a socially responsible reputation means that a brand consist of a wide range of social activities, including corporate philanthropy, community volunteering, and cause-related marketing (Biehal and Sheinin, 2007; Kim et al., 2012). As a result, eight brand reputation events were generated, of which all controlled four Chinese characters. The four CSR events were learning assistance activity, poverty alleviation program, welfare charity, and donation plan, while the CA events were technology development, technological innovation, technical seminar, and quality production. In addition, 50 people who did not participate in the formal ERPs experiment were queried by a 7-point Scale (1 = extremely disagree, 7 = extremely agree) whether the above mentioned events could be considered as the descriptions of CSR (*m* = 6.035 ± 1.012) and CA (*m* = 6.271 ± 1.214) reputation.

In the experiment, each set of stimuli used two verbs to connect among the brand, reputation event, and product, presenting in the form of one sentence (Peng et al., 2015) with the following five parts: brand (1) + verb (2) + reputation event (3) + launched (4) + product (5). The second verbs, respectively, matched with the following CSR or CA reputation event (e.g., “Yili was devoted to student supporting activity” and “Yili was devoted to technology development”). The stimuli sets with four conditions presented in Chinese characters (see Table 1). Each part of the stimulus group was presented on 300 × 200 pixels pictures. As a result, a total of 192 stimuli sets were contained in the entire experiment and assigned pseudorandomly to four blocks, with each block holding 48 trials. During the experiment, each participant was informed about taking a break for 5 min among the four blocks.

**TABLE 1:** Examples of the stimuli sets with four conditions.

**Conditions**	**Brand**	**Verb**	**Reputation event**	**Launched**	**Product**
CSR-high fit	Yili was devoted to student supporting activity and launched milk		
	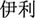	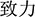	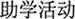	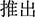	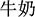
CA-high fit	Yili was devoted to technology development and launched milk		
	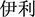	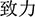	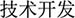	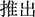	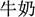
CSR-low fit	Yili initiated poverty alleviation program and launched T-shirt		
	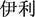	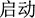	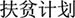	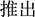	T 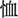
CA-low fit	Yili initiated technological innovation and launched T-shirt		
	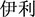	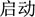	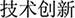	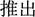	T 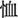

### Procedure

**FIGURE 1:**
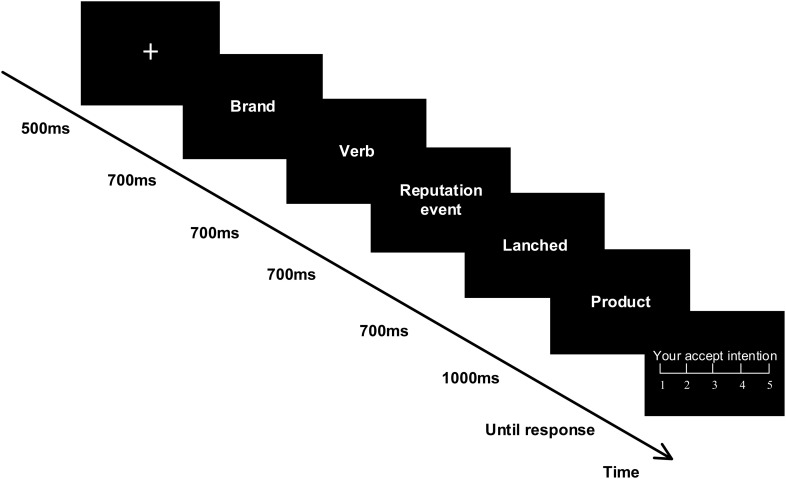
Experimental task: Participants were presented with four conditions of stimuli sets, each formed into one sentence in Chinese. They were instructed to report their acceptance intentions toward the given extension products on a five-point scale.

Participants were comfortably seated in a sound-attenuating room with a 23-inch computer screen (1,024 × 768 pixels, 60 HZ) approximately placed 70 cm in front of them. Prior to the formal experiment, they were given the experimental instructions and performed eight training trials as practice. As mentioned above, each stimulus set with a formation of one sentence consisted of five parts. Each part of the stimuli sets was presented at the center of the screen with a visual angle of 6.11°×3.05°. As Figure 1 illustrated, a fixation cross was first presented centrally on the screen against a black background for 500 ms, followed by a blank screen for 500 ms. Then, the sequential five parts of the stimuli were shown in order, and a random interval separately lasted for 500–800 ms among them. The first four parts, respectively, appeared for 700 ms, and the following product stimuli presented for 1,000 ms. At the end of each product display interface, a black screen was presented for 500 ms followed by a 5-point scale (1 = low accept intention to 5 = high accept intention) to examine the acceptance level of participants toward brand extension. The rating scale remained on the screen until they feedback. Participants were required to indicate their AI of the extension product by pressing the buttons (1, 2, 3, 4, 5) on the mini keypad. The presentation of stimuli and collection of behavioral reactions were achieved by applying the E-Prime 2.0 software (Psychology Software Tools, Pittsburgh, PA, United States).

### EEG Recordings and Analyses

Electroencephalogram (EEG) signals were recorded continuously using an electric cap with 64 Ag/AgCl electrodes in accordance with the standard international 10–20 system. The EEGs were sampled at 500 Hz with a 0.05–100 Hz bandpass filter using a Brain actiCHamp amplifier (Brain Products GmbH, Munich, Germany). Cz was selected as the online reference during recording, and the average of the left and right mastoids (TP9 and TP10) was computed as the offline reference. To measure the electrooculograms (EOGs), the electrodes were separately placed with supraorbital and infraorbital locations (vertical EOG) and the outer canthi of both eyes (horizontal EOG). The scalp impedances were maintained at less than 10 kΩ.

For the behavioral and ERP data, we conducted for all four conditions: CSR reputation with high fit brand extension (CSR-high fit), CSR reputation with low fit brand extension (CSR-low fit), CA reputation with high fit brand extension (CA-high fit), and CA reputation with low fit brand extension (CA-low fit). A two brand reputation event (CSR vs. CA) × two extension fit (high fit vs. low fit) ANOVA analysis was calculated for participants’ average AIs of brand extension to analyze the behavioral data. If there was an interaction effect between the two factors, a simple effect analysis was conducted.

The EEG recordings were analyzed offline through BrainVision Analyzer 2.1 software. Ocular artifacts, such as eye blink, were identified and removed using the independent component analysis (ICA) method (Semlitsch et al., 1986). The EEG recordings were segmented from 200 ms before the onset of product presentation to 800 ms after this onset, with 200 ms pre-target as the baseline. Moreover, any trials exceeding ± 100 V were excluded from the analysis. The EEG epochs were averaged separately for every participant in the following four conditions: CSR-high fit (*M* = 39.700, *SD* = 3.881), CSR-low fit (*M* = 40.300, *SD* = 4.835), CA-high fit (*M* = 40.500, *SD* = 3.606), and CA-low fit (*M* = 40.550, *SD* = 4.957). The ERPs data were filtered using a low-pass filter at 30 Hz (24 dB/Octave).

Based on the observation of the grand average of waveforms and the scalp distribution in our study, we analyzed two ERR components, namely, P2 and LPP. We chose the time window of 150 ms to 250 ms for P2 and 400 ms to 600 ms for LPP. Moreover, according to previous neuroscience studies (Dickter and Bartholow, 2007; Wang et al., 2012a), nine electrodes (FC1, FCz, FC2, C1, Cz, C2, CP1, CPz, CP2) were distributed among the frontal-central and central area and included in the statistical analysis for P2. We carried out a 2 (brand reputation event) × 2 (extension fit) × 6 (Electrode) three-way repeated-measures ANOVA test for the P2 analyses. Similarly, for the analysis of LPP, 9 electrodes (C1, Cz, C2, CP1, CPz, CP2, P1, Pz, P2) over the central-parietal area (Hua et al., 2014; Jin et al., 2017; Ma et al., 2018) were examined. We conducted a 2 × 2 × 9 ANOVA analysis for the LPP amplitude. When the interaction effect was notable, a simple effects analysis was operated. And the Greenhouse-Geisser correction (Greenhouse and Geisser, 1959) was utilized for correcting violations of the sphericity assumption. Spearman correlation analyses between the P2 and LPP amplitude were conducted.

## Results

### Behavioral Results

**FIGURE 2:**
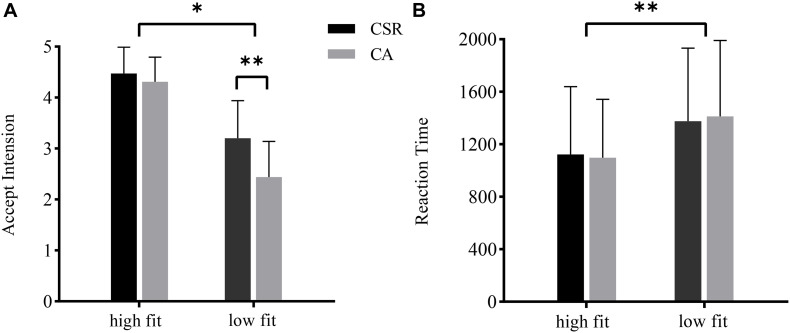
Behavioral results of the accept intention **(A)** and reaction time **(B)** for the four conditions of brand extension evaluation. ^∗^*p* < 0.05; ^∗∗^*p* < 0.01.

Two-way 2 (brand reputation event: corporate social responsibility vs. corporate ability) × 2 (extension fit: high fit vs. low fit) repeated-measures ANOVAs were performed for the AI and reaction time (RT). For the AI, there was a significant effect of the brand reputation [*F*_(1, 19)_ = 16.722, *p* < 0.01, η*_*p*_*^2^ = 0.468] and extension fit [*F*_(__1,__19)_ = 134.617, *p* < 0.001, η_*p*_^2^ = 0.876], and the CSR reputation (*M* = 3.836, *SE* = 0.122) had a higher AI than CA reputation (*M* = 3.373, *SE* = 0.108), the high fit conditions (*M* = 4.390, *SE* = 0.093) had a higher AI than low fit conditions (*M* = 2.820, S.E. = 0.144). The interaction effect between the brand reputation event and extension fit was notable [*F*_(1, 19)_ = 17.558, *p* < 0.001, η*_*p*_*^2^ = 0.480]. Therefore, a simple effect analysis was conducted (as shown in Figure 2). Under the condition of high fit, no significant effect was found between the CSR and CA reputation (*p* > 0.05). However, under the condition of low fit, the difference between the CSR and CA reputation was significant [*F*_(1, 19)_ = 28.450, *p* < 0.001, η*_*p*_*^2^ = 0.600], suggesting that consumers had a higher AI when a brand presented with CSR reputation (*M* = 3.202, *SE* = 0.165) than CA reputation (*M* = 2.438, *SE* = 0.157).

Regarding the RT, there were statistically significant differences between the high fit and low fit extension products [*F*_(__1,__19)_ = 37.768, *p* < 0.001, η*_*p*_*^2^ = 0.665], and the high fit conditions (*M* = 1110.146, *SE* = 106.096) had shorter RT than the low fit conditions (*M* = 1393.583, *SE* = 124.272) (as shown in Figure 2). There was no significant difference between the CSR and CA reputation, and no significant interaction effect was observed between brand reputation and extension fit.

### ERP Results

**FIGURE 3:**
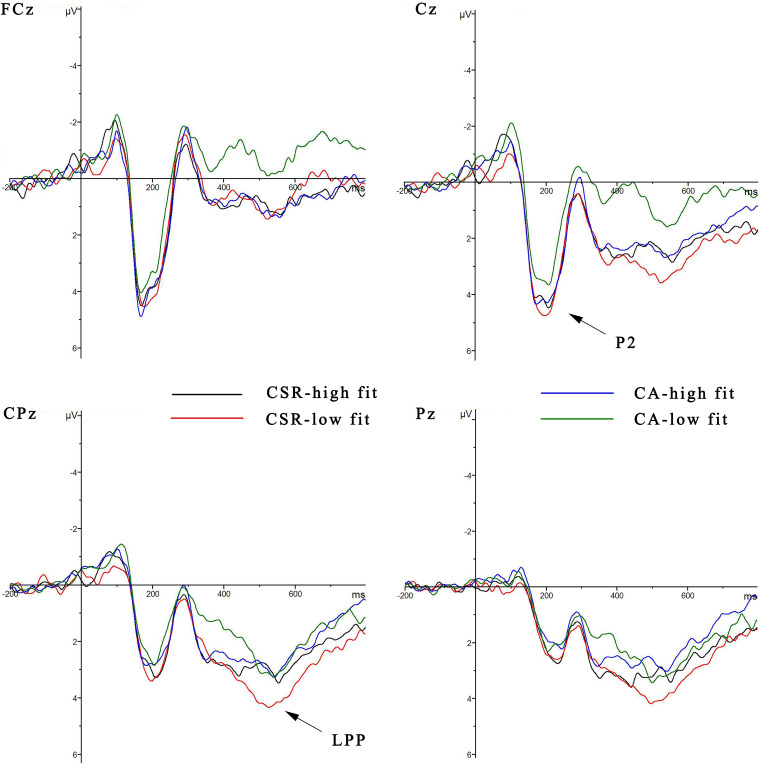
Grand averaged ERPs of P2 and LPP for brand reputation and perceived fit at midline electrodes (FCz, Cz, CPz, and Pz).

**FIGURE 4:**
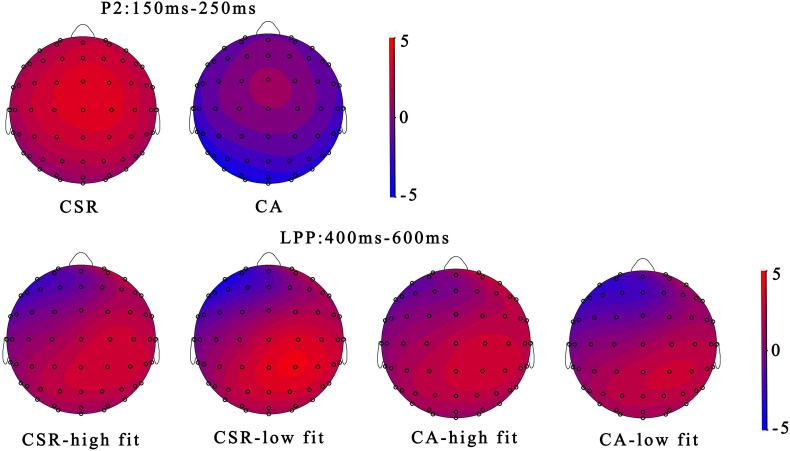
The scalp topographic distributions of the conditions at the P2 time window of 150–250 ms and LPP time window of 400–600 ms.

The grand-average ERPs waveforms and the topographic maps are shown in Figures 3, 4. A three-way 2 (brand reputation event: corporate social responsibility vs. corporate ability) × 2 (extension fit: high fit vs. low fit) × 9 (electrode:, FC1, FCz, FC2, C1, Cz, C2, CP1, CPz, and CP2) ANOVA was performed to analyze the P2 component in the time window of 150–250 ms. There was significant main effect of the brand reputation [*F*_(1, 19)_ = 4.478, *p* < 0.05, η*_*p*_*^2^ = 0.191]. And the CSR (*M* = 3.171, *SE* = 0.638) reputation evoked a larger P2 amplitude than CA (*M* = 2.786, *SE* = 0.640). However, the extension fit (*p* > 0.1), electrode (*p* > 0.1) and the interaction effect between the brand reputation and extension fit was not notable (*p* > 0.1).

A 2 (brand reputation event: CSR vs. CA) × 2 (extension fit: high fit vs. low fit) × 9 (electrode: C1, Cz, C2, CP1, CPz, CP2, P1, Pz, and P2) ANOVA was conducted to analyze the LPP component in the time window of 400–600 ms. No significant main effect of extension fit (*p* > 0.1) was found, but the brand reputation event [*F*_(1, 19)_ = 6.673, *p* < 0.05, η*_*p*_*^2^ = 0.260] was significant, reflecting that the CSR reputation (*M* = 2.898, *SE* = 0.548) evoked a larger LPP amplitude than CA reputation (*M* = 2.262, *SE* = 0.522). The interaction effect between brand reputation and extension fit was also significant [*F*_(1, 19)_ = 5.753, *p* < 0.05, η*_*p*_*^2^ = 0.232]. Therefore, a simple effect analysis was conducted. For the CSR reputation, there was a significant effect of extension fit [*F*(1, 19) = 4.750, *p* = 0.042, η*_*p*_*^2^ = 0.200], with the low fit condition (*M* = 3.227 μV, *SE* = 0.570) showing a larger LPP mean amplitude compared to the high fit condition (*M* = 2.569 μV, *SE* = 0.567). However, for the CA reputation, the mean amplitude of LPP between the high fit and low fit was not significantly different [*F*_(1, 19)_ = 0.723, *p* > 0.1, η*_*p*_^2^* = 0.037].

A correlation analysis between the amplitudes of P2 and LPP was conducted. The results showed that the P2 amplitude on FCZ was positively related to the LPP amplitude on Cz (*r* = 0.495, *p* < 0.001), CPz (*r* = 0.469, *p* < 0.001), and Pz (*r* = 0.362, *p* < 0.01).

## Discussion

In the current research, we explored the neurophysiological evidence of the interactive effects of brand reputation and perceived fit factors on the brand extension by using ERPs measurement. With the developing of brand competition in the marketplace, underling only one factor (such as perceived fit) on brand extension is not enough. The brand reputation, as a crucial determinant of driving corporate performance, should be highlighted in terms of brand extension literature. Thus, we added the brand reputation factor into previous brand extension studies by Ma et al. (2007, 2008; 2014). To be specific, we examined the consumers’ cognitive process when a brand with CSR or CA reputation extends to high fit or low fit extension product. The results of the experiment contribute to a better understanding of how the two types of brand reputation (CSR and CA) influence consumers’ brand extension evaluation.

With respect to behavioral results, the AI was higher in high fit conditions than low fit conditions. It has been widely proved that a higher perceived fit between the brand and extension product could help consumers to form a more positive attitude on extension evaluation (Boush and Loken, 1991; Smith and Andrews, 1995; Sichtmann and Diamantopoulos, 2013). Similarly, consumers were more inclined to accept a new product of the category near the original brand in the present experiment. As for the brand reputation events, participants held stronger AI for CSR rather than CA reputation. But it was only occurred in low fit conditions. In other words, the AIs of CSR and CA reputation were not different in high fit conditions. More deeply, for high fit extension products, there are both positive information for consumers whatever a brand with CSR or CA reputation. Thus they tended to positively evaluate high fit extension products with a brand with good reputations. In contrast, it is normally considered relatively impossible for the low fit extension products. When consumers faced a brand with an ability reputation, they firstly associated the quality of the brand’s existing products and largely believed that the brand could do well in operating products within their main business range. As for a brand with a CSR reputation, previous studies have provided evidence that corporate would gain favorable attitudes and positive support behaviors of consumers through engaging in social responsibility activities (Du et al., 2010). In the current study, when participants were attached with a brand with CSR reputation events, they might initially perceived kindness brand image. And then, if a new product of the brand appeared, they would intend to give a supporting to the brand which has engaged in social charity activities and further inclined to accept the new product. Accordingly, consumers gave a higher AI under the low fit conditions in the context of CSR than CA reputation.

Moreover, the reaction time (RT) was longer under low fit rather than high fit conditions. Incoherence with the previous brand extension study (Ma et al., 2008), the participants reacted slower for low fit than high fit conditions. As the low fit extension products are rarely seen under the umbrella brands, consumers need more cognitive efforts to consider the AI level of the clothes product information with the dairy brands. However, the main effect of the brand reputation and the interactive effect between the two factors were not significant in this study.

Neurophysiologically, the P2 component is related to early rapid automatic attention and it can be elicited by emotional stimuli, reflecting preliminary evaluation of affectively notable stimuli (Herbert et al., 2006; Olofsson et al., 2008). As mentioned in the introduction, previous scholars have verified that consumers could distinguish the emotionally positive degree of stimuli and show more attention to relatively positive stimuli (Jing et al., 2019). In the current study, the main effect of the two types of brand reputation (CSR and CA) was significant. Specifically, the P2 amplitude was larger under CSR reputation than CA reputation. As the building of brand reputations can assist consumers in generating a series of impressions or evaluations (Pritchard and Wilson, 2017), we interpreted that consumers could initially form a brand impression when they contacted with the CSR and CA events. It is noted that a brand with a CSR reputation generally associates with pro-social and other-oriented activities, such as welfare charity, providing a warm perception of consumers (Branco and Rodrigues, 2006; Kervyn et al., 2012; Johnson et al., 2019). And although the two types of brand reputation events were positive stimuli for subjects, a warm clue could augment a consumer’s emotional response compared to the competence perceptive (Xue et al., 2020). In addition, previous studies indicated that P2 could strongly respond to preference products or brands, reflecting more attentional resource engagement (Guo et al., 2016, 2018; Ma et al., 2018). Likewise, in our study, compared with the brand that only underlined its own competence, consumers might first form a warmth perception when a brand engaged in CSR activities and further improved their good impressions of the brand. Taken together, at the early stage of evaluation, consumers might hold more positive emotional value and preference toward the brand under CSR conditions and further engage more attention resources.

However, different from Ma et al.’s (2014) study, no significant distinction between the high fit and the low fit condition was found. The distinct results between our study and the previous study may be caused by a different paradigm. While the previous study used the S1 (brand name)-S2 (product name) paradigm, we adopted a new paradigm that contained the brand name, brand reputation event, and extension product, constructing a form of one sentence for participants’ better understanding. For more details, the participants first received the brand name with a reputation event, and then they were presented with the product name. They might first build the brand impression by exposure to CSR or CA reputation instead of rapidly connecting the relationship between the brand and the new product at the early phase. Therefore, the perceived fit of the brand extension was not processed during this stage.

During the late stage of brand extension evaluation, we observed the main effect of brand reputation and the interactive effect between brand reputation and perceived fit, reflecting different late positive potential (LPP) amplitude. For more details, a brand with a CSR reputation elicited a significantly larger LPP amplitude compared to that with a CA reputation. Prior studies have indicated that LPP is elicited by high emotional arousal and motivational significance stimuli (Hajcak et al., 2006; Fan et al., 2020). In our study, even though the two types of brand reputation events were both positive affect stimuli for participants, LPP responded more strongly to a brand with CSR than CA reputation. As previously mentioned, while the CSR activity is always considered as pro-social and moral and recruited more positive affect value, the CA activity just objectively reflects the brand itself competence and product quality. In addition, several lines of neural evidence have shown that prosocial products or brands could gain preference and good brand attitude of consumers, and augment brain activations associated with emotional awareness processing (Lee, 2016; Lee et al., 2020). Thereby, we suggested that a brand with CSR reputation which induced more attention and preference might arouse larger emotional motivations than CA reputation. Furthermore, under the CA reputation events, there was no significant LPP effect on the high fit and low fit conditions. However, the LPP was larger when subjects were exposed to a low fit condition in the context of CSR reputation (relative to CA reputation). The result was different from the previous brand extension study by Song et al. (2020), which discovered that high fit conditions between the luxury brand-service pairs could evoke greater arousing affect compared to low fit ones, as reflected by a greater LPP. Previous studies also pronounced that the LPP component is sensitive to moral or pro-social actions (Yoder and Decety, 2014). Although the CSR activity shows the pro-social behavior of a brand, we assumed that the effect would be enlarged under low fit brand extension conditions. To be specific, the low fit extension products as new categories of brands’ main products could largely stimulate consumers’ willingness to help toward a brand with a CSR reputation compared to the high fit conditions. Moreover, the formation of assisting intention toward low fit extension products might be attributed to the reciprocal altruism theory, which suggests that people are motivated to reciprocate the help providers when the latter need assistance (Trivers, 1971). Accordingly, when the consumers were exposed to the distant extension product of a pro-social brand, they might be altruistically motivated to help the brand extend its business range. Together, different from the previous brand extension study which indicated the affect transfer of the luxury brand extension, the LPP result of the present study might reflect emotional arousal and altruistic motivation in particular toward distant extension product.

In sum, our findings highlight the importance of brand reputation effect on brand extension. And we found two neuropsychological indicators (P2 and LPP), associated with attentional and emotional mechanisms, to assess CSR or CA reputation how to influence brand extension evaluation. Furthermore, the findings extend prior brand extension literature, which merely focused on one determinate factor of perceived fit. Our findings revealed the interaction effect of the two types of brand reputation (CSR vs. CA) and two fit levels (high fit vs. low fit) on brand extension evaluation. For more details, a brand with a CSR reputation elicited a larger P2 amplitude than a CA reputation. The consumers might form a warmer brand image and pay more attention toward a brand with CSR than CA activities at the early stage. Moreover, the LPP amplitude was larger under low fit conditions than high fit conditions in the context of CSR activities. Based on reciprocal altruism theory (Trivers, 1971), as a brand with a CSR reputation provides a kind image for consumers, we infer that low fit extension product might motivate people in return to help the brands to extend their business range. Thereby, the altruistic motivation was evoked during the late stage. Besides, our study contributes to providing neural evidence for managers to make efficient brand strategies to assist brands in launching new products in the competitive market.

The present study also has some limitations. For example, we only considered one category of original brands (dairy brands) as the research object and found that it is beneficial for these brands to engage in CSR activities. Yet, for prestige brands, such as luxury brands, it is possible to be confronted with a risk for their CSR disclosures (Janssen et al., 2013). Moreover, researchers have shown that the consumers’ cognitive process of brand extension toward basic and luxury brands is distinct (Song et al., 2020). Future studies should investigate the two types of brand reputation how to influence luxury brand extension. Besides, the sample size of the current study was not large, even though it is analogous with previous brand extension studies in the neuroscience domain by Jin et al. (2015); Fudali-Czyz et al. (2016), and Yang et al. (2018). It has been taken evidence that consumers with differences in demographic factors or personal traits respond to brand reputation in different ways (Bhaduri and Ha-Brookshire, 2015; Pérez and Rodríguez, 2017). Future measures should enlarge the sample size of subjects, including various characteristics of people, such as gender or empathy level, to replicate our research.

## Conclusion

The current study primarily investigated the consumers’ cognitive process toward the joint effects of brand reputation and perceived fit factors on the brand extension using the ERPs approach. The behavioral results demonstrated that consumers were intended to accept the high fit extension product. But if they were faced with distant extension products, they preferred the new product of a brand with CSR rather than CA reputation. At the neural level, CSR reputation aroused a larger P2 amplitude and LPP amplitude than CA reputation. Moreover, for a brand with a CSR reputation, low fit conditions elicited a more positive LPP amplitude than the high fit conditions. In contrast, for a brand with a CA reputation, the effect of perceived fit was not significant. The results might reflect early attentional resources engagement and altruistic motivation at the late stage. In general, a brand linked to CSR activities might augment the concern about the low fit extension product and further strengthen consumers’ altruistic motivation to help the brands broaden their business range. The findings are beneficial to make appropriate brand extension strategies for merchants in the actual marketplace.

## Data Availability Statement

The original contributions presented in the study are included in the article/Supplementary Material, further inquiries can be directed to the corresponding author/s.

## Ethics Statement

The studies involving human participants were reviewed and approved by the Internal Review Board of Yanshan University. The ethics committee waived the requirement of written informed consent for participation.

## Author Contributions

ZS and CL conceived and designed the experiment. CL and RS performed the experiment, analyzed the data, wrote, and edited the manuscript. All authors contributed to the revision of the article.

## Conflict of Interest

The authors declare that the research was conducted in the absence of any commercial or financial relationships that could be construed as a potential conflict of interest.

## Publisher’s Note

All claims expressed in this article are solely those of the authors and do not necessarily represent those of their affiliated organizations, or those of the publisher, the editors and the reviewers. Any product that may be evaluated in this article, or claim that may be made by its manufacturer, is not guaranteed or endorsed by the publisher.

## References

[B1] Aaker D. A. Keller K. L. (1990). Consumer evaluations of brand extensions. *J. Mark.* 54 27–41. 10.2307/1252171

[B2] Aaker J. L. Garbinsky E. N. Vohs K. D. (2012). Cultivating admiration in brands: warmth, competence, and landing in the “golden quadrant”. *J. Consum. Psychol.* 22 191–194. 10.1016/J.JCPS.2011.11.012

[B3] Bernat E. Bunce S. Shevrin H. (2001). Event-related brain potentials differentiate positive and negative mood adjectives during both supraliminal and subliminal visual processing. *Int. J. Psychophysiol.* 42 11–34. 10.1016/S0167-8760(01)00133-7 11451477

[B4] Bhaduri G. Ha-Brookshire J. (2015). Gender differences in information processing and transparency: cases of apparel brands’ social responsibility claims. *J. Prod. Brand Manag.* 24 504–517. 10.1108/jpbm-08-2014-0683

[B5] Biehal G. J. Sheinin D. A. (2007). The influence of corporate messages on the product portfolio. *J. Mark.* 71 12–25. 10.1509/jmkg.71.2.12 11670861

[B6] Bosshard S. S. Bourke J. D. Kunaharan S. Koller M. Walla P. Heinonen J. (2016). Established liked versus disliked brands: brain activity, implicit associations and explicit responses. *Cogent. Psychol.* 3:1176691. 10.1080/23311908.2016.1176691

[B7] Boush D. M. Loken B. (1991). A process-tracing study of brand extension evaluation. *J. Mark. Res.* 28 16–28. 10.2307/3172723

[B8] Boyd B. K. Bergh D. D. Ketchen D. J. (2009). Reconsidering the reputation-performance relationship: a resource-based view. *J. Manag.* 36 588–609. 10.1177/0149206308328507

[B9] Branco M. C. Rodrigues L. L. (2006). Corporate social responsibility and resource-based perspectives. *J. Bus. Ethics* 69 111–132. 10.2307/25123942

[B10] Brown T. J. Dacin P. A. (1997). The company and the product: corporate associations and consumer product responses. *J. Mark.* 61 68–84. 10.2307/1252190

[B11] Carretiea L. Mercado F. Tapiaa M. Hinojosa J. A. (2000). Emotion, attention, and the ‘negativity bias’, studied through event-related potentials. *Int. J. Psychophysiol.* 41 75–85. 10.1016/S0167-8760(00)00195-1 11239699

[B12] Couwenberg L. E. Boksem M. A. S. Dietvorst R. C. Worm L. Verbeke W. J. M. I. Smidts A. (2017). Neural responses to functional and experiential ad appeals: explaining ad effectiveness. *Int. J. Res. Mark.* 34 355–366. 10.1016/j.ijresmar.2016.10.005

[B13] Crowley K. E. Colrain I. M. (2004). A review of the evidence for P2 being an independent component process: age, sleep and modality. *Clin. Neurophysiol.* 115 732–744. 10.1016/j.clinph.2003.11.021 15003751

[B14] Czellar S. (2003). Consumer attitude toward brand extensions: an integrative model and research propositions. *Int. J. Res. Mark.* 20 97–115. 10.1016/s0167-8116(02)00124-6

[B15] Dickter C. L. Bartholow B. D. (2007). Racial ingroup and outgroup attention biases revealed by event-related brain potentials. *Soc. Cogn. Affect. Neurosci.* 2 189–198. 10.1093/scan/nsm012 18985140 PMC2569810

[B16] Du S. Bhattacharya C. B. Sen S. (2010). Maximizing business returns to corporate social responsibility (csr): the role of csr communication. *Int. J. Manag. Rev.* 12 8–19. 10.1111/j.1468-2370.2009.00276.x

[B17] Eren-Erdogmus I. Akgun I. Arda E. (2018). Drivers of successful luxury fashion brand extensions: cases of complement and transfer extensions. *J. Fash. Mark. Manag.* 22 476–493. 10.1108/jfmm-02-2018-0020

[B18] Fan B. Li C. Jin J. (2020). The brand scandal spillover effect at the country level: evidence from event-related potentials. *Front. Neurosci.* 13:1426. 10.3389/fnins.2019.01426 32038135 PMC6985369

[B19] Fombrun C. J. Gardberg N. A. Sever J. M. (2000). The reputation quotient: a multi-stakeholder measure of corporate reputation. *J. Brand Manag.* 7 241–255. 10.1057/bm.2000.10

[B20] Fombrun C. Shanley M. (1990). What’s in a name? Reputation building and corporate strategy. *Acad. Manage. J.* 33 233–258. 10.2307/256324

[B21] Fudali-Czyz A. Ratomska M. Cudo A. Francuz P. Kopis N. Tuznik P. (2016). Controlled categorisation processing in brand extension evaluation by Indo-European language speakers. An ERP study. *Neurosci. Lett.* 628 30–34. 10.1016/j.neulet.2016.06.005 27289044

[B22] Gonzalez-Villar A. J. Trinanes Y. Zurron M. Carrillo-de-la-Pena M. T. (2014). Brain processing of task-relevant and task-irrelevant emotional words: an ERP study. *Cogn. Affect. Behav. Neurosci.* 14 939–950. 10.3758/s13415-013-0247-6 24481851

[B23] Greenhouse S. W. Geisser S. (1959). On methods in the analysis of profile data. *Psychometrika* 24 95–112. 10.1007/BF02289823

[B24] Guo F. Ding Y. Wang T. Liu W. Jin H. (2016). Applying event related potentials to evaluate user preferences toward smartphone form design. *Int. J. Ind. Ergon.* 54 57–64. 10.1016/j.ergon.2016.04.006

[B25] Guo F. Wang X.-S. Liu W.-L. Ding Y. (2018). Affective preference measurement of product appearance based on event-related potentials. *Cogn. Technol. Work.* 20 299–308. 10.1007/s10111-018-0463-5

[B26] Hajcak G. Moser J. S. Simons R. F. (2006). Attending to affect: appraisal strategies modulate the electrocortical response to arousing pictures. *Emotion* 6 517–522. 10.1037/1528-3542.6.3.517 16938092

[B27] Handy T. C. Smilek D. Geiger L. Liu C. Schooler J. W. (2010). ERP evidence for rapid hedonic evaluation of logos. *J. Cognit. Neurosci.* 22 124–138. 10.1162/jocn.2008.21180 19199410

[B28] Herbert C. Kissler J. Junghofer M. Peyk P. Rockstroh B. (2006). Processing of emotional adjectives: evidence from startle EMG and ERPs. *Psychophysiology* 43 197–206. 10.1111/j.1469-8986.2006.00385.x 16712590

[B29] Herr P. M. Farquhar P. H. Fazio R. H. (1996). Impact of dominance and relatedness on brand extensions. *J. Consum. Psychol.* 5 135–159. 10.1207/s15327663jcp0502_03 26627889

[B30] Hua M. Han Z. R. Chen S. Yang M. Zhou R. Hu S. (2014). Late positive potential (LPP) modulation during affective picture processing in preschoolers. *Biol. Psychol.* 101 77–81. 10.1016/j.biopsycho.2014.06.006 25025638

[B31] Hur W.-M. Kim H. Woo J. (2013). How CSR leads to corporate brand equity: mediating mechanisms of corporate brand credibility and reputation. *J. Bus. Ethics* 125 75–86. 10.1007/s10551-013-1910-0

[B32] Ivens B. S. Leischnig A. Muller B. Valta K. (2015). On the role of brand stereotypes in shaping consumer response toward brands: an empirical examination of direct and mediating effects of warmth and competence. *Psychol. Mark.* 32 808–820. 10.1002/mar.20820

[B33] Janssen C. Vanhamme J. Lindgreen A. Lefebvre C. (2013). The catch-22 of responsible luxury: effects of luxury product characteristics on consumers’ perception of fit with corporate social responsibility. *J. Bus. Ethics* 119 45–57. 10.1007/s10551-013-1621-6

[B34] Jin J. Wang C. Yu L. Ma Q. (2015). Extending or creating a new brand: evidence from a study on event-related potentials. *Neuroreport* 26 572–577. 10.1097/WNR.0000000000000390 26053698

[B35] Jin J. Zhang W. Chen M. (2017). How consumers are affected by product descriptions in online shopping: event-related potentials evidence of the attribute framing effect. *Neurosci. Res.* 125 21–28. 10.1016/j.neures.2017.07.006 28734975

[B36] Jing K. Mei Y. Song Z. Wang H. Shi R. (2019). How do price and quantity promotions affect hedonic purchases? An ERPs study. *Front. Neurosci.* 13:526. 10.3389/fnins.2019.00526 31231177 PMC6558398

[B37] Johnson Z. S. Lee Y. J. Ashoori M. T. (2017). Brand associations: the value of ability versus social responsibility depends on consumer goals. *J. Brand Manag.* 25 27–37. 10.1057/s41262-017-0070-4

[B38] Johnson Z. S. Mao H. Lefebvre S. Ganesh J. (2019). Good guys can finish first: how brand reputation affects extension evaluations. *J. Consum. Psychol.* 29 565–583. 10.1002/jcpy.1109

[B39] Jung N. Y. Seock Y.-K. (2016). The impact of corporate reputation on brand attitude and purchase intention. *Fash. Text.* 3:20. 10.1186/s40691-016-0072-y

[B40] Kervyn N. Fiske S. T. Malone C. (2012). Brands as intentional agents framework: how perceived intentions and ability can map brand perception. *J. Consum. Psychol.* 22 166–176. 10.1016/j.jcps.2011.09.006 24403815 PMC3882007

[B41] Kim Y. Park M. S. Wier B. (2012). Is earnings quality associated with corporate social responsibility? *Account. Rev.* 87 761–796. 10.2308/accr-10209

[B42] Lee E.-J. (2016). Empathy can increase customer equity related to pro-social brands. *J. Bus. Res.* 69 3748–3754. 10.1016/j.jbusres.2015.05.018

[B43] Lee E.-J. Choi H. Han J. Kim D. H. Ko E. Kim K. H. (2020). How to “Nudge” your consumers toward sustainable fashion consumption: an fMRI investigation. *J. Bus. Res.* 117 642–651. 10.1016/j.jbusres.2019.09.050

[B44] Liao W. Zhang Y. Peng X. (2019). Neurophysiological effect of exposure to gossip on product endorsement and willingness-to-pay. *Neuropsychologia* 132:107123. 10.1016/j.neuropsychologia.2019.107123 31207265

[B45] Liu B. Xin S. Jin Z. Hu Y. Li Y. (2010). Emotional facilitation effect in the picture-word interference task: an ERP study. *Brain Cogn.* 72 289–299. 10.1016/j.bandc.2009.09.013 19853986

[B46] Luck S. J. Woodman G. F. Vogel E. K. (2000). Event-related potential studies of attention. *Trends Cogn. Sci.* 4 432–440. 10.1016/S1364-6613(00)01545-X 11058821

[B47] Ma H. Mo Z. Zhang H. Wang C. Fu H. (2018). The temptation of zero price: event-related potentials evidence of how price framing influences the purchase of bundles. *Front. Neurosci.* 12:251. 10.3389/fnins.2018.00251 29731705 PMC5919942

[B48] Ma Q. Wang C. Wang X. (2014). Two-stage categorization in brand extension evaluation: electrophysiological time course evidence. *PLoS One* 9:e114150. 10.1371/journal.pone.0114150 25438152 PMC4250186

[B49] Ma Q. Wang M. Da Q. (2020). The effects of brand familiarity and product category in brand extension: an ERP study. *Neurosci. Res.* 169 48–56. 10.1016/j.neures.2020.06.010 32652108

[B50] Ma Q. Wang X. Dai S. Shu L. (2007). Event-related potential N270 correlates of brand extension. *Neuroreport* 18 1031–1034. 10.1097/wnr.0b013e3281667d59 17558290

[B51] Ma Q. Wang X. Shu L. Dai S. (2008). P300 and categorization in brand extension. *Neurosci. Lett.* 431 57–61. 10.1016/j.neulet.2007.11.022 18155837

[B52] Ma Y. Jin J. Yu W. Zhang W. Xu Z. Ma Q. (2018). How is the neural response to the design of experience goods related to personalized preference? An implicit view. *Front. Neurosci.* 12:760. 10.3389/fnins.2018.00760 30416423 PMC6214219

[B53] Medina C. A. G. Martínez-Fiestas M. Aranda L. A. C. Sánchez-Fernández J. (2021). Is it an error to communicate CSR Strategies? Neural differences among consumers when processing CSR messages. *J. Bus. Res.* 126 99–112. 10.1016/j.jbusres.2020.12.044

[B54] Olofsson J. K. Nordin S. Sequeira H. Polich J. (2008). Affective picture processing: an integrative review of ERP findings. *Biol. Psychol.* 77 247–265. 10.1016/j.biopsycho.2007.11.006 18164800 PMC2443061

[B55] Ozdemir S. Zhang S. Gupta S. Bebek G. (2020). The effects of trust and peer influence on corporate brand-consumer relationships and consumer loyalty. *J. Bus. Res.* 117 791–805. 10.1016/j.jbusres.2020.02.027

[B56] Parker J. R. Lehmann D. R. Keller K. L. Schleicher M. G. (2017). Building a multi-category brand: when should distant brand extensions be introduced? *J. Acad. Mark. Sci.* 46 300–316. 10.1007/s11747-017-0552-7

[B57] Peng X. Li Y. Wang P. Mo L. Chen Q. (2015). The ugly truth: negative gossip about celebrities and positive gossip about self entertain people in different ways. *Soc. Neurosci.* 10 320–336. 10.1080/17470919.2014.999162 25580932

[B58] Pérez A. Rodríguez I. (2017). Personal traits and customer responses to CSR perceptions in the banking sector. *Int. J. Bank Mark.* 35 128–146. 10.1108/ijbm-02-2016-0023

[B59] Pritchard M. Wilson T. (2017). Building corporate reputation through consumer responses to green new products. *J. Brand Manag.* 25 38–52. 10.1057/s41262-017-0071-3

[B60] Ramesh K. Saha R. Goswami S. Dahiya R. (2019). Consumer’s response to CSR activities: mediating role of brand image and brand attitude. *Corp. Soc. Responsib. Environ. Manag.* 26 377–387. 10.1002/csr.1689

[B61] Sattler H. Völckner F. Riediger C. Ringle C. M. (2010). The impact of brand extension success drivers on brand extension price premiums. *Int. J. Res. Mark.* 27 319–328. 10.1016/j.ijresmar.2010.08.005

[B62] Schupp H. T. Cuthbert B. N. Bradley M. M. Cacioppo J. T. Ito T. Lang P. J. (2000). Affective picture processing: the late positive potential is modulated by motivational relevance. *Psychophysiology* 37 257–261. 10.1111/1469-8986.3720257 10731776

[B63] Seghier M. L. (2013). The angular gyrus: multiple functions and multiple subdivisions. *Neuroscientist* 19 43–61. 10.1177/1073858412440596 22547530 PMC4107834

[B64] Semlitsch H. V. Anderer P. Schuster P. Presslich O. (1986). A solution for reliable and valid reduction of ocular artifacts, applied to the P300 ERP. *Psychophysiology* 23 695–703. 10.1111/j.1469-8986.1986.tb00696.x 3823345

[B65] Shang Q. Pei G. Dai S. Wang X. (2017). Logo effects on brand extension evaluations from the electrophysiological perspective. *Front. Neurosci.* 11:113. 10.3389/fnins.2017.00113 28337121 PMC5341626

[B66] Sichtmann C. Diamantopoulos A. (2013). The impact of perceived brand globalness, brand origin image, and brand origin-extension fit on brand extension success. *J. Acad. Mark. Sci.* 41 567–585. 10.1007/s11747-013-0328-7

[B67] Smith D. C. Andrews J. (1995). Rethinking the effect of perceived fit on customers’ evaluations of new products. *J. Acad. Mark. Sci.* 23 4–14. 10.1007/BF02894607

[B68] Song Z. Liu C. Shi R. Zhang M. Wang H. Mei Y. (2020). Neural activities during the evaluation of luxury goods-to-service brand extension: an event-related potentials (ERPs) study. *J. Neurosci. Psychol. Econ.* 13 127–140. 10.1037/npe0000132

[B69] Swaminathan V. (2003). Sequential brand extensions and brand choice behavior. *J. Bus. Res.* 56 431–442. 10.1016/s0148-2963(01)00242-9

[B70] Trivers R. L. (1971). The evolution of reciprocal altruism. *Q. Rev. Biol.* 46 35–57. 10.1086/406755

[B71] Völckner F. Sattler H. (2006). Drivers of brand extension success. *J. Mark.* 70 18–34. 10.1509/jmkg.70.2.18 11670861

[B72] Wang D. H.-M. Chen P.-H. Yu T. H.-K. Hsiao C.-Y. (2015). The effects of corporate social responsibility on brand equity and firm performance. *J. Bus. Res.* 68 2232–2236. 10.1016/j.jbusres.2015.06.003

[B73] Wang H. Song Z. Shi R. Mei Y. Liu C. (2019). How expertise congruency effect matters in celebrity/brand endorsements: electrophysiological time course evidence. *Neurosci. Lett.* 712:134436. 10.1016/j.neulet.2019.134436 31479725

[B74] Wang X. Huang Y. Ma Q. Li N. (2012a). Event-related potential P2 correlates of implicit aesthetic experience. *Neuroreport* 23 862–866. 10.1097/WNR.0b013e3283587161 22922601

[B75] Wang X. Ma Q. Wang C. (2012b). N400 as an index of uncontrolled categorization processing in brand extension. *Neurosci. Lett.* 525 76–81. 10.1016/j.neulet.2012.07.043 22884930

[B76] Xue J. Zhou Z. Zhang L. Majeed S. (2020). Do brand competence and warmth always influence purchase intention? The moderating role of gender. *Front. Psychol.* 11:248. 10.3389/fpsyg.2020.00248 32153466 PMC7046750

[B77] Yang T. Kim S. P. (2019). Group-level neural responses to service-to-service brand extension. *Front. Neurosci.* 13:676. 10.3389/fnins.2019.00676 31316343 PMC6610219

[B78] Yang T. Lee S. Seomoon E. Kim S. P. (2018). Characteristics of human brain activity during the evaluation of service-to-service brand extension. *Front. Hum. Neurosci.* 12:44. 10.3389/fnhum.2018.00044 29479313 PMC5811480

[B79] Yoder K. J. Decety J. (2014). Spatiotemporal neural dynamics of moral judgment: a high-density ERP study. *Neuropsychologia* 60 39–45. 10.1016/j.neuropsychologia.2014.05.022 24905282 PMC4104265

[B80] Zhang W. Jin J. Wang A. Ma Q. Yu H. (2019). Consumers’ implicit motivation of purchasing luxury brands: an EEG study. *Psychol. Res. Behav. Manag.* 12 913–929. 10.2147/prbm.s215751 31576184 PMC6768311

[B81] Zhang Y. Kwak H. Puzakova M. Taylor C. R. (2020). When distraction may be a good thing: the role of distraction in low-fit brand extension evaluation. *Psychol. Mark.* 37 604–621. 10.1002/mar.21329

[B82] Zheng X. Baskin E. Dhar R. (2019). By-brand or by-category? The effect of display format on brand extension evaluation. *J. Retail.* 95 76–85. 10.1016/j.jretai.2019.04.003

[B83] Zubair M. Iqbal S. Usman S. M. Awais M. Wang R. Wang X. (2020). Message framing and self-conscious emotions help to understand pro-environment consumer purchase intention: an ERP study. *Sci. Rep.* 10:18304. 10.1038/s41598-020-75343-8 33110155 PMC7591878

